# Identifying patients with chronic hepatitis B at high risk of type 2 diabetes mellitus: a cross-sectional study with pair-matched controls

**DOI:** 10.1186/s12876-015-0263-9

**Published:** 2015-03-19

**Authors:** Yi Shen, Jian Zhang, Hui Cai, Jian-Guo Shao, You-Yi Zhang, Yan-Mei Liu, Gang Qin, Yan Qin

**Affiliations:** 1 Department of Epidemiology and Medical Statistics, School of Public Health, Nantong University, Nantong, Jiangsu 226019 China; 2 Center for Liver Diseases, Nantong Third People’s Hospital, Nantong University, Nantong, Jiangsu 226006 China; 3 Department of Internal Medicine, Singapore General Hospital, Singapore, 169608 Singapore

**Keywords:** Chronic hepatitis B, Type 2 diabetes mellitus, Cross-sectional study

## Abstract

**Background:**

The presence of diabetes mellitus (DM) is associated with increased liver morbidity and mortality risk in patients with chronic hepatitis B (CHB). Aim of this study was to identify factors associated with type 2 diabetes mellitus (T2DM) in CHB patients.

**Methods:**

A cross-sectional study with pair-matched controls was conducted in Nantong Third People’s Hospital, Nantong University, China. From January 2008 to December 2012, a total of 1783 CHB patients were screened for study subjects, among whom 207 patients with T2DM were enrolled as cases and 207 sex- and age-matched non-DM patients as controls. Demographic, anthropometric, lifestyle, clinical, and laboratory data were obtained from each subject.

**Results:**

In the univariate model, thirteen variables showed marked differences between the DM group and non-DM group. Patients with longer duration of CHB (≥15 years) and alcoholic steatosis showed the highest likelihood of T2DM (odds ratio = 5.39 and 4.95; 95% confidence intervals 2.76-10.53 and 1.65-14.91). In the multivariate adjusted analysis, three CHB-related factors, namely high viral load, long duration of illness, and presence of cirrhosis, contributed to substantially increase the likelihood of T2DM, in addition to the other five risk factors including family history of DM, low education level, elevated triglycerides (TG), gamma-glutamyl transferase (GGT) levels, and presence of alcoholic steatosis.

**Conclusions:**

Our findings suggest that high viral load, long duration of CHB, presence of cirrhosis, alcoholic steatosis and several other factors may be potential risk factors for development of T2DM in CHB patients. It is of vital importance to monitor glucose in high-risk CHB patients and aggressively intervene on modifiable risk factors.

## Background

Hepatitis B virus (HBV) infection is a major global public health issue with increasing complication and mortality rates. As a highly endemic country, China bears an estimated 93 million chronic HBV carriers and 30 million chronic hepatitis B (CHB) patients [1]. Approximately 300,000 patients die from HBV-related liver diseases each year in China [2]. Abnormalities in glucose metabolism are common in patients with chronic liver diseases. The presence of diabetes mellitus (DM) and poor diabetic control is associated with increased liver morbidity and mortality risk in patients with HBV-cirrhosis [3]. Type 2 diabetes mellitus (T2DM) is independently associated with the increased risk of hepatocellular carcinoma (HCC) in CHB patients [4-6]. Although it is not clear whether HBV infection has a relation with the development of diabetes [7,8], identifying patients at high risk of diabetes and improving diabetic control should be essential part of the good care for the CHB patients.

While epidemiological studies have evaluated factors associated with the presence of T2DM in general population, the risk factors among CHB patients has not been explored. The aim of this hospital-based cross-sectional study was to identify risk factors associated with type 2 diabetes in patients with chronic hepatitis B.

## Methods

### Study population

The CHB patients with T2DM (DM group) that formed the basis of this study comprised all patients who fulfilled the following criteria: (1) admitted to Nantong Third People’s Hospital, Nantong University (Jiangsu Province, China) between 1 January 2008 and 31 December 2012; (2) diagnosed as chronic hepatitis B or hepatitis B cirrhosis, without evidence of viral hepatitis other than hepatitis B; (3) complicated with newly diagnosed or previously known T2DM; (4) duration of diabetes not longer than that of hepatitis B surface antigen (HBsAg) positivity. Non-DM controls were patients with CHB who had never been diagnosed with diabetes, and they were matched for sex and age with DM patients at a ratio of 1:1.

Chronic hepatitis B was diagnosed according to the practice guideline of the American Association for the Study of Liver Diseases [9]. All patients were known to have positive hepatitis B surface antigen (HBsAg) for more than six months. Diagnostic criteria for diabetes mellitus include the following: symptoms of diabetes plus casual plasma glucose concentration ≥ 11.1 mmol/L (200 mg/dL); or fasting plasma glucose (FPG) levels ≥ 7.0 mmol/L (126 mg/dL) on 2 separate occasions; or a 2-h postload glucose ≥11.1 mmol/L during an oral glucose tolerance test (OGTT) on 2 separate occasions [10,11]. The use of the hemoglobin A1c (HbA1c) for the diagnosis of diabetes has not been recommended in China [11]. Non-invasive evaluation of fatty liver (steatosis) and cirrhosis was performed with ultrasound, computed tomography scan or transient elastography FibroTouch (Wuxi Hisky Medical Technology, Beijing, China). The diagnosis of alcoholic steatosis was made by documentation of alcohol excess and evidence of fatty liver [12].

This study protocol conformed to the ethical guidelines of the 1975 Declaration of Helsinki and was duly approved by the ethics committee of Nantong Third People’s Hospital, Nantong University. Written informed consents for inclusion in the study were obtained from all patients.

### Data collection

The interview included questions related to the diagnosis and treatment of diabetes and hepatitis. A standard questionnaire was administered by trained staff to obtain the following information: age (year), sex, body mass index (BMI; weight [kg]/height [m^2^]), systolic and diastolic blood pressure (mmHg), marital status (yes/no), educational level (primary/under, middle/high school, college/above), history of CHB and T2DM (yes/no), date of diagnosis of CHB and T2DM, family history of CHB and T2DM (yes/no, first-degree relatives), alcohol consumption (none; moderate, <30/20 g/d men/women; or excessive, ≥ 30/20 g/d men/women) and smoking habit (none; moderate, < 10 cigarettes/d; or excessive, ≥ 10 cigarettes/d).

Blood samples were collected from all subjects at time of enrollment in study, after at least 8 h of fasting. FPG, HbA1c, serum alanine aminotransferase (ALT), aspartate transaminase (AST), bilirubin (TBIL), gamma-glutamyltransferase (GGT), albumin (ALB), prothrombin time (PT), triglycerides (TG), cholesterol (Chol), high density lipoprotein-cholesterol (HDL), low density lipoprotein cholesterol (LDL) and creatinine (CREA) were measured by biochemical tests using an automatic biochemical analyzer (AU2700, Olympus, Japan). Fasting plasma insulin (FPI) concentration was measured by two-site immunoenzymometric assay using Roche Elecsys 2010 autoanalyzer (Roche Diagnostics, USA). Serum alpha-fetoprotein (AFP) levels were measured by an immunoluminometric assay on a random-access analyser (Architect i2000; Abbott Diagnostics, USA). Serological tests for HBsAg and HBeAg and the quantification of HBV DNA load were determined as we described elsewhere [13]. Serological tests for anti-HAV IgM, anti-HCV, anti-HDV and anti-HEV were performed using enzyme immunoassay methods.

### Statistical analysis

The continuous data were expressed as mean ± standard deviation (SD) and categorical data as number or percent. Comparison of continuous variables was done by student t test. For categorical variables the chi-square or Fisher’s exact test were used. In this analysis, T2DM was taken as the dependent variable. Demographic, lifestyle, medical history, clinical and laboratory factors were taken as independent variables. Risk factors found significant on univariate analysis were entered into multivariate logistic regression model. All statistical tests were 2-tailed, and a significance level (*P*) of 0.05 was used. The statistical tests were performed using SPSS for Windows version 20 (Chicago, IL).

## Results

During the study period, a total of 1,783 patients were admitted to our hospital with the diagnosis of CHB. After excluding patients who were co-infected with other hepatitis viruses, 1732 patients were recruited for the study. Their mean age was 45.8 ± 15.11 years, and 82.8% were men. There were 1343 CHB patients with no evidence of cirrhosis, and 389 patients with hepatitis B cirrhosis.

**Figure 1:**
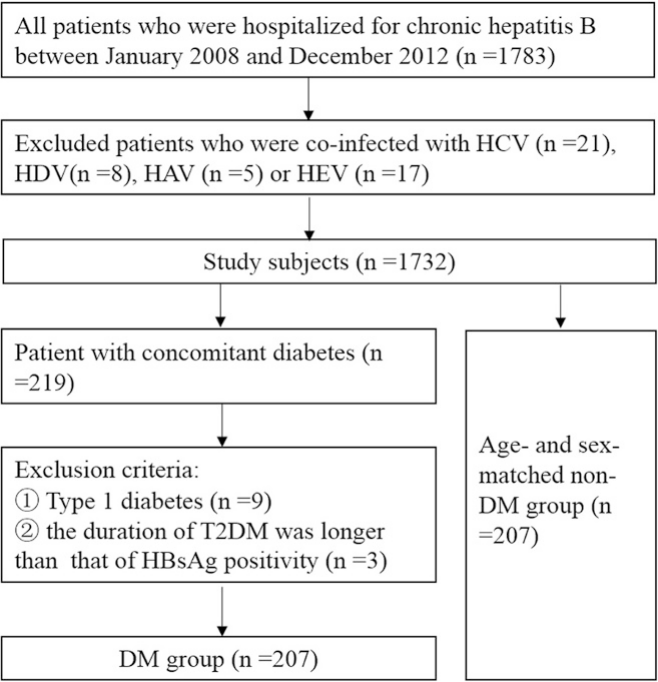
**Flowchart of selection of study patients.**

The diagnosis of diabetes mellitus was made for 219 CHB patients (49 newly diagnosed), among whom 207 patients met the inclusion criteria for the DM group. At the index date, 207 non-DM controls were pair-matched by sex and year of birth (Figure 1).

### Characteristics of demographics, lifestyle, and medical history

As shown in Table 1, we observed a significant difference of education level, history of smoking and alcohol drinking, hypertension, duration of CHB, family history of CHB and family history of DM between the DM and non-DM groups (*P <* 0.05). Patients with T2DM had longer CHB duration (9.26 ± 8.78 vs. 4.97 ± 6.22 years, *P <* 0.001). The percentages of smoking or drinking history were significantly higher among the diabetic patients when compared to the non-diabetic patients. As expected, the diabetic patients also had more often a diagnosis of hypertension (20.29% vs. 10.63%, *P <* 0.01). No significant difference was found on BMI and marital status between the two groups.

**Table 1:** **Characteristics of demographic, lifestyle and medical history**

	DM (n = 207)	Non-DM (n = 207)	*P*-value
Sex (Male/Female)	163/44	163/44	-
Age	51.34 ± 10.87	50.16 ± 10.49	0.262
< 40	34	33	
40-59	118	124	
≥ 60	55	43	0.469
BMI (kg/m^2^)	25.55 ± 4.30	24.86 ± 3.82	0.085
< 18.5	18	22	
18.5-23.9	73	85	
24-27.9	72	67	
≥ 28	44	33	0.382
Education			
Primary/under	106	73	
Middle/High school	91	120	
College/above	10	14	0.005
Marital status			
Yes	189	196	
No^a^	18	11	0.178
History of smoking			
No	132	171	
Yes	75	36	< 0.001
History of alcohol drinking			
No	138	174	
Yes	69	33	< 0.001
Hypertension			
No	165	185	
Yes	42	22	0.007
Family history of CHB			
No	175	192	
Yes	32	15	0.008
Duration of CHB (year)	9.26 ± 8.78	4.97 ± 6.22	< 0.001
< 5	97	158	
5-9.9	41	22	
10-14.9	26	14	
≥ 15	43	13	< 0.001
Family history of DM			
No	187	199	
Yes	20	8	0.019
Duration of DM (year)			
0^b^	49 (23.67%)		
0.1-1.9	99 (47.83%)		
2-4.9	46 (22.22%)		
5-9.9	10 (4.83%)		
≥ 10	3 (1,45%)		

^a^including divorced, widowed and unmarried.

^b^newly-diagnosed.

The proportion of patients with newly-diagnosed DM, duration less than 2 years, 2 to 4.9 years, 5 to 9.9 years, or more than 10 years, was 24%, 48%, 22%, 5% and 1% respectively.

### Clinical and laboratory features

The percentage of HBeAg-positive CHB patients was significantly higher in the DM group than that in the non-DM group (45.89% vs. 31.88%, *P <* 0.01). The mean HBV DNA level was higher in the DM group, compared with the non-DM group (5.34 ± 1.59 vs. 4.78 ± 1.96 lg copies/mL, *P <* 0.01). Concerning the mean levels of serum ALT, AST, TBIL, ALB, PT and AFP, there were no significant differences found between the two groups. However, for serum GGT and creatinine, the DM patients had higher levels than the non-DM controls. The mean levels of plasma triglyceride, FPG, HbA1c and insulin were significantly higher in the DM group than in the non-DM group, whereas the HDL cholesterol level was lower in the DM patients than in the non-DM controls (Table 2).

**Table 2:** **Clinical and laboratory features**

	DM (n = 207)	Non-DM (n = 207)	*P*-value
ALT (U/L)	315.54 ± 435.76	245.23 ± 343.80	0.069
AST (U/L)	201.43 ± 272.55	172.34 ± 250.14	0.259
TBIL (μmol/L)	70.86 ± 83.05	57.34 ± 78.99	0.090
ALB (g/L)	34.21 ± 10.45	35.98 ± 9.57	0.073
PT (s)	16.51 ± 6.15	15.48 ± 5.94	0.083
GGT (U/L)	175.11 ± 162.11	121.12 ± 131.38	< 0.001
AFP (ng/mL)	64.96 ± 115.44	56.29 ± 118.27	0.451
HBeAg positivity	95 (45.89%)	66 (31.88%)	0.003
HBV DNA (lg copies/mL)	5.34 ± 1.59	4.78 ± 1.96	0.002
CREA (μmol/L)	82.32 ± 49.62	69.03 ± 38.21	0.002
TG (mmol/L)	1.84 ± 0.93	1.22 ± 0.62	< 0.001
Chol (mmol/L)	4.25 ± 3.06	3.84 ± 2.27	0.122
HDL (mmol/L)	1.08 ± 0.55	1.27 ± 0.69	0.002
LDL (mmol/L)	2.34 ± 1.31	2.10 ± 1.52	0.086
FPG (mmol/L)	8.67 ± 4.20	5.11 ± 1.28	< 0.001
HbA1c (%)	6.81 ± 2.07	6.12 ± 1.48	< 0.001
FPI (μU/mL)	9.42 ± 7.78	6.26 ± 3.33	< 0.001
Steatosis	28 (13.53%)	10 (4.83%)	< 0.001
Cirrhosis	51 (24.64%)	34 (16.43%)	0.040
HCC (%)	12 (5.35%)	7 (3.21%)	0.240
NA treatment^a^	79 (38.16%)	73 (35.27%)	0.541
DM treatment			
Diet alone	62 (29.95%)		
Metformin	54 (26.09%)		
Sulphonylurea	19 (9.18%)		
Insulin	72 (34.78%)		

^a^NA: nucleos(t)ide analogue.

Reference range of variables: ALT, 0-40 U/L; AST, 0-40 U/L; TBIL, 3.4-17.1 μmol/L; ALB, 35-55 g/L; PT, 12.7-15.4 s; GGT, 0-50U/L; AFP, 0-13.4 ng/mL; HBV DNA, <3 lg copies/mL); CREA, 44-115 μmol/L; TG, 0.48-1.88 mmol/L; Chol, 3.2-6.5 mmol/L; HDL, 0.83-1.96 mmol/L; LDL, 0-3.36 mmol/L; FPG, 3.9-5.6 mmol/L; HbA1c, 4.0-6.0%; FPI, 2-20 μU/mL.

The higher prevalence rates of steatosis and cirrhosis were found in the DM group than the non-DM group (13.53% vs. 4.83%, 24.64% vs. 16.43%, *P <* 0.05). The DM group did not differ in the percentage of HCC from the non-DM group (Table 2).

Antiviral therapy with nucleos(t)ide analogues (NAs) was either initiated or continued in 38.16% (79/207) of the DM patients and 35.27% (73/207) of the non-DM patients (*P* = 0.55) at the time of enrollment. Entecavir was the most common NA used at baseline diagnosis in 41 (19.81%) and 32 (15.46%), lamivudine in 24 (11.59%) and 15 (7.25%), telbivudine in 3 (1.45%) and 10 (4.83%), and lamivudine plus adefovir in 11 (5.31%) and 6 (2.90%) of DM and non-DM patients respectively. Of the DM patients, 62 (29.95%) had diet-controlled DM, 72 (34.78%) were on insulin, 54 (26.09%) on metformin, and 19 (9.18%) patients on sulphonylurea treatment.

### Factors associated with T2DM in patients with CHB

A univariate logistic analysis was performed to quantify the effect of potential risk factors on T2DM in CHB patients and generated the odds ratio (OR) and 95% confidence interval (CI). Long duration of CHB (≥ 15 years) and concomitant alcoholic steatosis were the two most important and significant risk factors with OR (95% CI) of 5.39 (2.76-10.53) and 4.95 (1.65-14.91) respectively. The logistic regression analysis showed that high viral load (HBV DNA ≥ 10^6^copies/mL) significantly increased the OR for developing type 2 diabetes (OR 2.84, 95% CI 1.73-4.66; *P <* 0.001), as did the presence of cirrhosis (OR 1.66, 95% CI 1.02-2.70; *P <* 0.05), HBeAg positivity (OR 1.81, 95% CI 1.21-2.70; *P <* 0.01), and family history of CHB (OR 2.36, 95% CI 1.18-4.71; *P <* 0.05). Family history of DM, low education level, smoking and alcohol drinking habits, hypertension, high GGT and TG, and were also associated with the increased risk of T2DM. No significant effect of LDL level or presence of HCC on the risk of diabetes was observed (Table 3).

**Table 3:** **Univariate and multivariate-adjusted logistic analysis of risk factors of T2DM in CHB patients**

Variable	DM (n = 207)	Non-DM (n = 207)	Univariate		Multivariate adjusted	
			OR (95% CI)	*P*-value	OR (95% CI)	*P*-value
Education						
Primary/under	106	73	1		1	
Middle/High	91	120	0.52 (0.35-0.78)	0.002	0.60 (0.37-0.99)	0.046
College/above	10	14	0.49 (0.21-1.17)	0.108	0.54 (0.20-1.48)	0.231
Smoking						
None	132	171	1		1	
Moderate (< 10 cigarettes/d)	51	26	2.54 (1.50-4.29)	< 0.001	1.60 (0.79-3.27)	0.195
Excessive (≥ 10 cigarettes/d)	24	10	3.11 (1.44-6.73)	0.004	2.22 (0.91-5.34)	0.080
Alcohol Consumption						
None	138	174	1		1	
Moderate (< 30/20 g/d men/women)	29	19	1.92 (1.04-3.58)	0.039	1.64 (0.74-3.60)	0.222
Excessive (≥ 30/20 g/d men/women)	40	14	3.60 (1.88-6.89)	< 0.001	2.09 (0.90-4.83)	0.086
Hypertension						
No	165	185	1		1	
Yes	42	22	2.14 (1.23-3.74)	0.007	1.46 (0.74-2.88)	0.281
Family history of DM						
No	187	199	1		1	
Yes	20	8	2.66 (1.14-6.19)	0.023	3.85 (1.43-10.39)	0.008
Family history of CHB						
No	175	192	1		1	
Yes	32	15	2.34 (1.23-4.47)	0.010	1.45 (0.70-2.98)	0.314
Duration of CHB (year)						
<5	97	158	1		1	
5-9.9	41	22	3.04 (1.71-5.40)	< 0.001	4.32 (2.21-8.44)	< 0.001
10-14.9	26	14	3.03 (1.51-6.07)	0.002	2.13 (0.97-4.69)	0.061
≥15	43	13	5.39 (2.76-10.53)	< 0.001	5.80 (2.72-12.37)	< 0.001
GGT (U/L)						
<50	31	58	1		1	
50-199	96	106	1.69 (1.01-2.84)	0.045	1.54 (0.83-2.87)	0.174
≥200	80	43	3.48 (1.96-6.17)	< 0.001	3.79 (1.89-7.59)	< 0.001
HBeAg positive						
No	112	141	1		1	
Yes	95	66	1.81 (1.21-2.70)	0.004	1.33 (0.80-2.22)	0.275
HBV DNA (copies/mL)						
< 10^3^	38	68	1		1	
10^3^-9.9*10^5^	58	62	1.50 (0.89-2.55)	0.130	1.27 (0.66-2.43)	0.468
≥ 10^6^	111	77	2.84 (1.73-4.66)	< 0.001	1.94 (1.05-3.58)	0.033
High TG (≥ 1.7 mmol/L)						
No	144	181	1		1	
Yes	63	26	3.05 (1.84-5.05)	< 0.001	2.12 (1.15-3.90)	0.016
Low HDL (< 1.0 mmol/L)						
No	138	153	1			
Yes	69	54	1.42 (0.93-2.16)	0.107		
Steatosis						
No	179	197	1			
Non-alcoholic	10	6	1.83 (0.65-5.15)	0.249	1.28 (0.35-4.78)	0.708
Alcoholic	18	4	4.95 (1.65-14.91)	0.004	5.24 (1.50-18,29)	0.009
Cirrhosis						
No	156	173	1		1	
Yes	51	34	1.66 (1.02-2.70)	0.040	2.00 (1.11-3.61)	0.020
HCC						
No	195	200	1			
Yes	12	7	1.76 (0.68-4.56)	0.246		

In the multivariate adjusted model, T2DM remained significantly associated with lower education level, family history of DM, longer duration of CHB (≥ 15 years), higher viral load (HBV DNA ≥ 10^6^ copies/mL), presence of cirrhosis, elevated GGT level (≥ 200 U/L, upper limit of normal or ULN for 50 U/L), higher TG (≥ 1.7 mmol/L), and alcoholic steatosis, as shown in Table 3.

## Discussion

The liver plays an important role in the metabolism of carbohydrates and is responsible for balance of blood glucose [14]. In the presence of liver diseases, the metabolic homeostasis of glucose is often impaired [15]. The alleged mechanisms consist of insulin resistance (IR) in a hitherto unexplained way, direct pancreatic islet β-cell damage, perhaps caused by an autoimmune process via molecular mimicry or by dysregulation of autoimmune functions [16]. Moreover, the etiology of liver disease is important in the incidence of diabetes: the non-alcoholic fatty liver disease (NAFLD), alcohol and hepatitis C virus (HCV) are more frequently associated with diabetes [17-19].

Among the few previous studies about the relationship of HBV infection and diabetes, findings were controversial [7,8]. A positive association was found in a cohort study in which HBsAg-positive Asian American subjects had a higher risk of incident diabetes (OR 9.73; 95% CI, 3.30-28.69), compared with non-infected controls [7]. In contrast, in a 10-year Taiwanese community-based cohort study, persons with asymptomatic chronic HBV infection did not have an increased risk of diabetes, in comparison with non-HBV controls [8]. In this hospital-based cross-sectional study with pair-matched controls, CHB-related variables, namely duration of CHB, HBV viral load and cirrhosis were associated with patients with type 2 diabetes mellitus compared to those without.

IR, associated with impaired cellular response of the insulin signaling pathway, is a risk factor for T2DM. The association between CHB and IR also remains unclear [20-22]. In this study, we observed the hyperinsulinemia in diabetic CHB patients, suggesting the body responded by increasing serum insulin concentrations to compensate IR. BMI is supposed to be an important potential intermediary or confounder for diabetes risk association. But we observed no difference of the mean BMI between the DM group and non-DM group. It might be explained in part by the fact that Chinese develop DM at a notably lower BMI [23] and by the low rate of newly diagnosed DM in this study.

HBV infection status in these CHB patients was classified by HBeAg status and HBV DNA level in our study. Univariate analysis showed that the percentage of patients with positive HBeAg or high HBV DNA in the case group was significantly higher than that of control group. Multivariate-adjusted Logistic analysis further confirmed that high viral load (HBV DNA ≥ 10^6^copies/mL) was independently associated with T2DM in patients with CHB.

We noted a significant association between CHB duration and T2DM risk in our study population, a four-fold higher diabetes risk for subjects with CHB duration ≥ 5 years than for those with a shorter duration (< 5 years). A positive association between cirrhosis and diabetes risk was also observed. These results suggest that the association between CHB and diabetes risk may be a time- and severity-dependent relationship.

It is hard to accurately determine the severity of chronic hepatitis on the basis of physical examination and liver biochemistry. Liver enzymes ALT and GGT elevation have been reported to be independent predictors for diabetes in the general population [24]. In our stratified analysis, we found that the elevation of GGT (≥ 4 ULN), but not ALT, was an independent risk factor of diabetes after adjustment for other variables.

Fatty liver in HBV-infected patients seems to be as frequent as in the general population [20,25]. Glucose, the key component of diabetes, is overproduced by the fatty liver. In a previous nine-year study, the fatty liver index (FLI) for evaluating the extent of liver fat has been found predictive of incident diabetes [26]. In this study, alcoholic steatosis is seen more commonly in the DM patients. Both alcohol consumption and alcoholic steatosis increased the risk of diabetes in the univariate analysis. After multivariate adjustment, alcoholic steatosis remained to be an independent risk factor. This finding suggests that alcoholic liver disease plays an important role in the development of diabetes in relatively lean Chinese population.

When the family history of DM, a well-known risk factor for DM, was entered through logistic model, the correlation between CHB-related factors and DM remained significant. This finding, in consistence with previous studies [15], indicates that liver injury per se is associated with DM, while the family history of DM is only an adjunctive factor. As similar observations from general population [23], elevated serum triglyceride level and lower educational level were also significantly associated with an increased risk of diabetes among these CHB patients.

Although we were able to demonstrate a potential positive association with T2DM and three CHB-related factors, our study is hypothesis generating rather than proving a firm cause-effect relationship due to several limitations. It is apparent that such a cross-sectional study with pair-matched controls generally do not allow interpretation with respect to an etiological or causal relation. In an attempt to establish a temporal relationship, we aimed to assess exposure to CHB prior to onset of DM, implicating that this exposure might trigger some metabolic pathways for DM. Cirrhosis was identified as an independent risk factor for DM, we were only able to suggest a potential relationship between the severity of CHB and the incidence of DM. Besides, our patient population is a selected hospital-based population, which may not represent the CHB population as a whole. These problems could be addressed in a population-based cohort study which follow patients with HBV from time of infection for a long enough time to the incidence of diabetes.

## Conclusions

The findings of our study indicate that the risk of T2DM in CHB patients is not homogeneous and varied substantially due to the presence of quite a few potential diabetic risk factors. In addition to other risk factors, such as family history of diabetes, low education level, elevated serum GGT and TG level, presence of alcoholic steatosis, three CHB-related features, i.e. long duration of CHB, high HBV load and presence of cirrhosis, contribute to substantially increase the likelihood of T2DM. Despite its role as an independent predictor of cardiovascular, renal and hepatic outcomes, the importance of monitoring diabetes and acting on modifiable risk factors among CHB patients is still underestimated. Physicians should screen for diabetes in patients at a greater risk, in order to improve care, target lifestyle and medical interventions, and reduce the clinical and economic burden of both hepatic and diabetic complications.

## Abbreviations

CHB: Chronic hepatitis B
T2DM: Type 2 diabetes mellitus
HBV: Hepatitis B virus
DM: Diabetes mellitus
HCC: Hepatocellular carcinoma
HBsAg: Hepatitis B surface antigen
BMI: Body mass index
ALT: Alanine aminotransferase
AST: Aspartate transaminase
TBIL: Bilirubin
GGT: Gamma-glutamyltransferase
ALB: Albumin
PT: Prothrombin time
FPG: Fasting plasma glucose
HbA1c: Hemoglobin A1c
FPI: Fasting plasma insulin
TG: Triglycerides
Chol: Cholesterol
HDL: High density lipoprotein-cholesterol
LDL: Low density lipoprotein cholesterol
CREA: Creatinine
AFP: Alpha-fetoprotein
HBeAg: Hepatitis B e antigen
OR: Odds ratio
CI: Confidence interval
SD: Standard deviation
ULN: Upper limit of normal
IR: Insulin resistance
NAFLD: Non-alcoholic fatty liver disease
HCV: Hepatitis C virus

## References

[1] Lu FM, Zhuang H (2009). Management of hepatitis B in China. Chin Med J (Engl).

[2] Wang FS, Fan JG, Zhang Z, Gao B, Wang HY (2014). The global burden of liver disease: the major impact of China. Hepatology.

[3] Hsiang JC, Gane EJ, Bai WW, Gerred SJ (2015). Type 2 diabetes: a risk factor for liver mortality and complications in hepatitis B cirrhosis patients. J Gastroenterol Hepatol.

[4] Li Q, Li WW, Yang X, Fan WB, Yu JH, Xie SS (2012). Type 2 diabetes and hepatocellular carcinoma: a case–control study in patients with chronic hepatitis B. Int J Cancer.

[5] Lai MS, Hsieh MS, Chiu YH, Chen TH (2006). Type 2 diabetes and hepatocellular carcinoma: a cohort study in high prevalence area of hepatitis virus infection. Hepatology.

[6] Chen CL, Yang HI, Yang WS, Liu CJ, Chen PJ, You SL (2008). Metabolic factors and risk of hepatocellular carcinoma by chronic hepatitis B/C infection: a follow-up study in Taiwan. Gastroenterology.

[7] Li-Ng M, Tropp S, Danoff A, Bini E (2007). Association between chronic hepatitis B virus infection and diabetes among Asian Americans and Pacific Islanders. Dig Liver Dis.

[8] Huang ZS, Huang TS, Wu TH, Chen MF, Hsu CS, Kao JH (2010). Asymptomatic chronic hepatitis B virus infection does not increase the risk of diabetes mellitus: a ten‐year observation. J Gastroenterol Hepatol.

[9] Lok AS, McMahon BJ (2007). Chronic hepatitis B. Hepatology.

[10] American Diabetes A (2006). Diagnosis and classification of diabetes mellitus. Diabetes Care.

[11] Ji L-N, Lu J-M, Weng J-P, Jia W-P, Zhou Z-G, Zou D-J (2012). China guideline for type 2 diabetes. Chin J Diabetes.

[12] O'Shea RS, Dasarathy S, McCullough AJ, Practice Guideline Committee of the American Association for the Study of Liver D, Practice Parameters Committee of the American College of G (2010). Alcoholic liver disease. Hepatology.

[13] Qin G, Shao JG, Wang B, Shen Y, Zheng J, Liu XJ (2014). Artificial liver support system improves short- and long-term outcomes of patients with HBV-associated acute-on-chronic liver failure: a single-center experience. Medicine.

[14] Postic C, Dentin R, Girard J (2004). Role of the liver in the control of carbohydrate and lipid homeostasis. Diabetes Metab.

[15] Alavian SM, Hajarizadeh B, Nematizadeh F, Larijani B (2004). Prevalence and determinants of diabetes mellitus among Iranian patients with chronic liver disease. BMC Endocr Disord.

[16] Picardi A, D'Avola D, Gentilucci UV, Galati G, Fiori E, Spataro S (2006). Diabetes in chronic liver disease: from old concepts to new evidence. Diabetes Metab Res Rev.

[17] Tolman KG, Fonseca V, Dalpiaz A, Tan MH (2007). Spectrum of liver disease in type 2 diabetes and management of patients with diabetes and liver disease. Diabetes Care.

[18] Wang C-S, Wang S-T, Yao W-J, Chang T-T, Chou P (2007). Hepatitis C virus infection and the development of type 2 diabetes in a community-based longitudinal study. Am J Epidemiol.

[19] Thuluvath PJ, John PR (2003). Association between hepatitis C, diabetes mellitus, and race. a case–control study. Am J Gastroenterol.

[20] Wang CC, Hsu CS, Liu CJ, Kao JH, Chen DS (2008). Association of chronic hepatitis B virus infection with insulin resistance and hepatic steatosis. J Gastroenterol Hepatol.

[21] Lee JG, Lee S, Kim YJ, Cho BM, Park JS, Kim HH (2012). Association of chronic viral hepatitis B with insulin resistance. World J Gastroenterol.

[22] Tkachenko LI, Maleev VV (2014). Clinical features of chronic hepatitis B in the presence of metabolic syndrome and insulin resistance. Ter Arkh.

[23] Yang W, Lu J, Weng J, Jia W, Ji L, Xiao J (2010). Prevalence of diabetes among men and women in China. N Engl J Med.

[24] Doi Y, Kubo M, Yonemoto K, Ninomiya T, Iwase M, Tanizaki Y (2007). Liver enzymes as a predictor for incident diabetes in a Japanese population: the Hisayama study. Obesity.

[25] Machado MV, Oliveira AG, Cortez-Pinto H (2011). Hepatic steatosis in hepatitis B virus infected patients: meta-analysis of risk factors and comparison with hepatitis C infected patients. J Gastroenterol Hepatol.

[26] Balkau B, Lange C, Vol S, Fumeron F, Bonnet F, Group Study DESIR (2010). Nine-year incident diabetes is predicted by fatty liver indices: the French D.E.S.I.R. study. BMC Gastroenterol.

